# The wellbeing of adolescents and the role of greenness: A cross-sectional study among Italian students

**DOI:** 10.3389/fpubh.2022.1050533

**Published:** 2023-01-18

**Authors:** Giacomo Scaioli, Giulia Squillacioti, Michela Bersia, Valeria Bellisario, Alberto Borraccino, Roberto Bono, Paola Dalmasso, Patrizia Lemma

**Affiliations:** ^1^Department of Public Health and Pediatrics, University of Torino, Torino, Italy; ^2^Post Graduate School of Medical Statistics, University of Torino, Torino, Italy

**Keywords:** greenness, wellbeing, adolescents, urbanization, psychosomatic health complaints

## Abstract

**Introduction:**

Adolescence is a critical period of life, and the level of wellbeing acquired during this stage might have an influence on health status in adulthood. The wellbeing of adolescents is associated with both biological and environmental determinants. To date, few studies have evaluated the effect of exposure to urban green spaces (i.e., greenness) on adolescents' wellbeing. Therefore, the aim of this study is to assess the association between exposure to greenness and the wellbeing of adolescents, accounting for the level of urbanization surrounding schools.

**Methods:**

In the frame of the 2018 Italian Health Behaviour in School-aged Children (HBSC), we analyzed cross-sectional data from the Piedmont Region. Exposure to greenness was quantified by the Normalized Difference Vegetation Index (NDVI). Schools were geocoded, and a fixed buffer (radius 1,500 m) centered on each school was then built to enable average NDVI calculations. Adolescents' wellbeing was assessed by self-reported psychological, somatic, and psychosomatic health complaints as follows. Respondents were asked how often, in the last 6 months, they had experienced: (a) headache, (b) stomachache, (c) backache, (d) dizziness, (e) feeling low, (f) irritability or bad temper, (g) feeling nervous, and (h) difficulties getting to sleep using the HBSC Symptom Checklist (HBSC-SCL), an eight-item tool. Multivariable, multilevel logistic regression models tested the association between exposure to NDVI and psychosomatic, somatic, and psychological health complaints, one at a time, using schools as a random intercept.

**Results:**

In total, 2065 subjects (47.6% girls) aged 11 (48.4%) and 13 (51.6%) years were involved. Greenness was found to be inversely associated with reported psychosomatic (OR 0.72, 95% CI: 0.53–0.98) and psychological health complaints (OR 0.67, 95% CI: 0.49–0.92) in boys only, adjusting for age, urbanization level, and socioeconomic status, and stratifying by gender.

**Discussion:**

Our results support the implementation of future policies for urban environmental design supporting the increase of green spaces, as suggested by the United Nations Sustainable Development Goals. Further studies are needed to confirm our findings.

## 1. Introduction

Adolescence is a critical period of life, and the level of wellbeing acquired during this stage might have an influence on health status in adulthood (1, 2). Specifically, low levels of wellbeing in adolescents might be linked with an increased risk of the onset of cardiovascular diseases, elevated blood pressure, obesity, and smoking habits among teenagers, all conditions that persist later in life with age (1, 2). Given these premises, it is of utmost importance to investigate the wellbeing of young people and the factors potentially associated with higher or lower wellbeing levels.

Wellbeing is a multifaceted concept that can be described and measured in different ways (3–9). According to the Centers for Diseases Control and Prevention (CDCs), wellbeing “includes the presence of positive emotions and moods (e.g., contentment, happiness), and the absence of negative emotions (e.g., depression, anxiety), satisfaction with life, fulfillment, and positive functioning” (10). Wellbeing is further defined as a balance between psychological, social, and physical resources and challenges: if the former predominates, wellbeing levels are impaired (11).

An appropriate, indirect measure of the wellbeing of adolescents could be self-reported scales exploring a wide symptomatology, both psychological and somatic (4, 12, 13), previously investigated among adolescents (4, 14, 15). Self-reported measures are needed and highly recommended for this category of people because adolescents are usually in good health, and therefore, objective and instrument-measured health data are usually not available (4). In this regard, a recent worldwide meta-analysis showed a stable trend of psychosomatic health complaints since 2010 among adolescents (15). At the country level, Bersia et al. report an increasing trend of psychosomatic health complaints among adolescents in the last decade in Italy, especially for psychological complaints and among 13- to 15-year-old girls (4).

Regarding the potential determinants influencing the level of wellbeing of adolescents, previous studies have investigated the relationships between academic stress, social support (i.e., family, peers), violence exposure, socioeconomic status, and physical activity (4, 13, 16).

Recently, wellbeing was found to be related to contact with public green space, also known as “greenness.” Greenness, which has been defined as “green schoolyards” (17), is the availability of parks (green space) (18) and tree density (19), and the accessibility of and distance of residential areas from urban green spaces (20). Regardless of the adopted methodology, higher exposure to greenness has been associated with positive health outcomes, both mental and physical: improved mental health and reduced stress, lower body weight, reduced blood pressure, stronger immune system, decreased incidence of diabetes, overall lower mortality, and faster hospital recovery (21–23).

To the best of our knowledge, few studies have evaluated the effect of exposure to greenness on the mood, perceived stress, and resilience of adolescents (18, 24). These studies demonstrate that the percentage of park area within cities could be used to predict perceived stress levels (18) and that students in green schoolyards build competence and cooperative social relationships (17), which have positive protective effects on wellbeing. From a wider point of view, exposure to greenness could be significantly associated with improved adolescent mood (24). As the concept is relatively new, the current literature indicates the need for further evidence to better understand the relationship between greenness and wellbeing among adolescents.

Strictly related to greenness, the concept of urbanization as “a process that leads to the growth of cities due to industrialization and economic development and that leads to urban-specific changes in specialization, labor division, and human behaviors” (25) has increased worldwide in recent decades. According to World Bank data, from 1990 to 2020, the percentage of the world population living in urban areas increased from 43 to 56% (26), and it has been estimated that more than 80% of the global population will live in urban areas in 2050 (26). Although urbanization has been proven to have some positive effects on health, mainly due to easier access to healthcare services and higher education and career opportunities (27), this phenomenon might also impact levels of wellbeing. Previous studies investigating how the increased level of urbanization could modulate the level of wellbeing in adults and adolescents (28, 29) have found that urbanization significantly affects the subjective wellbeing of the general population only in some regions and areas (29). These results might have been due to the complex relationship between urbanization and wellbeing, which might also depend on factors such as the proximity of rural areas to large cities, economic factors, and the level of innovation in the area (28). Urbanization is also related to greenness; urban sprawl and/or increased population density have led to a reduction in green spaces (30) and citizens' access to green spaces and natural vegetation (31).

The present study aims to assess the potential relationship between greenness exposure and wellbeing in adolescents living in Piedmont, a northwestern region of Italy, taking into account the level of urbanization in the area surrounding their schools. The main hypothesis of this study is that higher exposure to greenness is associated with improved wellbeing in adolescents.

## 2. Methods

Data were collected as part of the Italian 2018 Health Behavior in School-aged Children (HBSC) study. The study is a World Health Organization (WHO) collaborative international project aiming to describe health-related attitudes and behaviors in the adolescent population (32). A detailed description of the international and Italian HBSC study protocol can be found elsewhere (33, 34).

As the primary sampling unit, schools were selected by systematic cluster sampling from a list of all public and private schools obtained from the Italian Ministry of Education, University, and Research. A representative sample of adolescents aged 11, 13, and 15 years (corresponding to the 6th, 8th, and 10th grades, respectively) for each of the Italian regions was invited to participate in the study. Data on the students' social background, health indicators, and health-related behaviors were collected in classroom settings using a standardized, anonymous, self-administered questionnaire.

According to the international protocol, Italian study procedures, methods, and questionnaires were formally approved by the Ethics Committee of the Italian National Institute of Health. Participation was voluntary and opt-out consent was obtained from the parents/caregivers of the students involved. The anonymity and confidentiality of all participants were ensured (32, 33).

The present study relies on data from Piedmont, a northwestern Italian region, the second largest region in Italy, with a population of more than four million inhabitants, nearly 550,000 of whom are of school age (Italian national statistical institute 2020) (35). For the present study, only data related to students aged 11 and 13 years were considered. Since greenness measures were collected at the school level, and since students aged 11 and 13 years attend school widely distributed within the whole Italian territory, unlike high schools, which are often far from the students' homes, the measurement of greenness in the surroundings of the schools attended by the students is a more reliable measure for those aged 11–13 years than for those aged 15 years.

### 2.1. Outcome variables

#### 2.1.1. Psychological and somatic health complaints

The HBSC Symptom Checklist (HBSC-SCL) is a measure composed of eight items (36, 37). The checklist shows adequate test-retest reliability and psychometric properties and has been extensively used to study adolescent mental wellbeing (36, 37). Respondents were asked to indicate how often in the last 6 months they had experienced: (a) headache, (b) stomachache, (c) backache, (d) dizziness, (e) feeling low, (f) irritability or bad temper, (g) feeling nervous, and (h) difficulties getting to sleep. Response options ranged from “about every day” to “rarely or never” for each symptom. These eight items, which are referred to as psychosomatic health complaints (PHC), can be further grouped into somatic (SOMHC: headache, stomachache, backache, and dizziness) and psychological health complaints (PSYHC: feeling low, irritability, feeling nervous, and sleeping difficulties).

According to the international report (38), a binary cut-off of the presentation of “at least two health complaints more than once a week” was used for somatic, psychological, and overall health complaints (39).

### 2.2. Potential determinants of the outcome variables

#### 2.2.1. Exposure to greenness

Exposure to greenness was evaluated through the Normalized Difference Vegetation Index (NDVI). All sampled schools were initially geolocalized, and a fixed buffer (radius 1,500 m) around each school was then built to allow for NDVI calculations. Schools were considered proxies of students' home addresses, given that adolescents of 11 and 13 years old usually attend schools near their homes and do not have independent mobility (40).

NDVI is a metric used to quantify vegetated biomass from satellite images, calculated by measuring the ratio of the difference between the near-infrared (NIR) and red light to their sum. This measure ranges from −1 to +1: higher positive values indicate the presence of active photosynthetic vegetation (healthy green vegetation). NDVI was assessed from cloud-free satellite images (Landsat 5), referring to the same year of sampling of the HBSC study (2018), during summer to capture the maximum vegetated canopy cover (41). A radius of 1,500 m was decided upon in order to consider all the green spaces that could be accessed by the students attending the school in question. Shorter radii could have led to an underestimation of the greenness around the schools included in the study (22).

### 2.3. Control variables and confounding factors

Control variables and potential confounding factors, namely urbanization, gender, age, and socioeconomic status (SES), were included.

#### 2.3.1. Urbanization

Urbanization is included in the model based on the Italian Institute of Statistics (ISTAT) classification (42). Since 2011, Eurostat (and therefore ISTAT) has classified municipalities into three different classes of urbanization (high, medium, low) using a tool based on population density and the number of inhabitants evaluated within regular grids with cells of one square kilometer (43).

#### 2.3.2. Socioeconomic status (SES)

SES was measured using the Family Affluence Scale (FAS), a six-item scale developed and validated within the HBSC study (44, 45). FAS is composed of six indicators of the SES of the family:

- Whether the family has a car, van, or truck;
- Whether the adolescent has their own bedroom;
- How many times the adolescent has traveled away on holiday with the family;
- How many computers the family owns;
- Ownership of a dishwasher; and
- The number of bathrooms in the adolescent's house.

The score ranges from 0 to 13. According to scientific literature, the scores of the responses to the four questions were summed up and divided into three groups: 0–6 (low), 7–9 (medium), and 10–13 (high) SES (45).

### 2.4. Statistical analysis

Categorical variables were described with percentages and absolute numbers, and continuous variables were described with means and standard deviations. Fisher's exact test or the Mann–Whitney test were used to compare boys and girls, as appropriate. Multivariable, multilevel logistic regression models were performed using “school” as the higher level and students as the lower level for the following outcome variables: (1) psychosomatic; (2) somatic; and (3) psychological health complaints. As independent variables, the NDVI (1,500 m) was considered a continuous variable showing the estimates as one-IQR increase. Moreover, given the interaction between gender and the health outcomes, all models were stratified for gender and adjusted for age group, SES, and urbanization. A two-tailed statistical significance level of 5% was set up. All analyses were performed using STATA v16.1 (StataCorp LLC: College Station, TX, USA).

## 3. Results

The overall regional sample included 2,065 subjects aged 11 (48.4%) and 13 (51.6%) years, of which 47.6% were girls. Within the sample, 48.3% of the adolescents declared at least two psychosomatic health complaints (PHC) more than once a week, while 18% (11.5% of boys and 25.3% of girls) and 38.3% (32.4% of boys and 44.6% of girls) presented at least two somatic (SOMHC) and psychological health complaints (PSYHC) more than once a week, respectively (Table 1).

**Table 1:** Description of the sample.

		**Boys (*n* = 1,082)**	**Girls (*n* = 983)**	**Total (*n* = 2,065)**	***p*-value**
Age category	11th year	522 (48.2%)	478 (48.6%)	1,000 (48.4%)	0.895
	13th year	560 (51.8%)	505 (51.4%)	1,065 (51.6%)	
At Least two somatic health complaints more than		124 (11.5%)	245 (25.3%)	369 (18.0%)	<0.001
once a week (SOMHC)					
At least two psychological health complaints more		348 (32.4%)	437 (44.6%)	785 (38.3%)	<0.001
than once a week (PSYHC)					
At least two psychosomatic health complaints more		437 (40.8%)	554 (56.7%)	991 (48.3%)	<0.001
than once a week (PHC)					
Normalized Difference Vegetation Index (NDVI)		0.52 (±0.15)	0.52 (±0.15)	0.52 (±0.15)	0.670
1,500 meters					
Urbanization density	Low	260 (24.1%)	219 (22.3%)	479 (23.2%)	0.640
	Medium	589 (54.4%)	546 (55.5%)	1,135 (54.9%)	
	High	233 (21.5%)	218 (22.2%)	451 (21.9%)	
Family affluence scale (FAS)	Low	241 (22.7%)	256 (26.4%)	497 (24.5%)	0.085
	Medium	514 (48.4%)	465 (48.0%)	979 (48.2%)	
	High	307 (28.9%)	248 (25.6%)	555 (27.3%)	

Results are expressed as percentages and absolute numbers, and NDVI 1,500 with means and standard deviations. *P*-values are calculated with Fisher's exact test or the Mann–Whitney test.

### 3.1. Univariable and multivariable, multilevel logistic regression analyses—Association between greenness and psychosomatic health complaints

**Figure 1:**
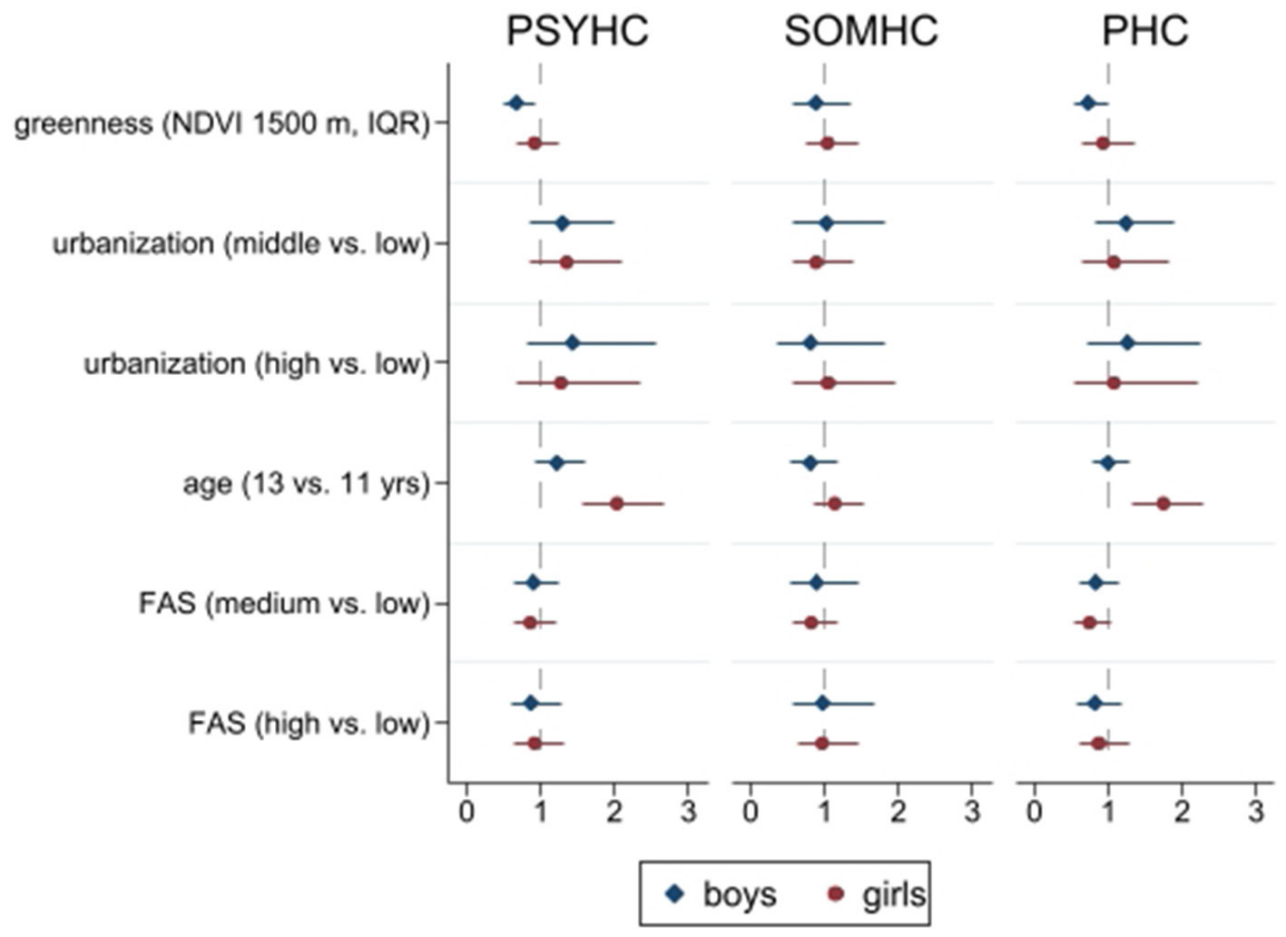
Multivariable multilevel regression models. Outcomes: PSYHC, Psychological Health Complaints; SOMHC, Somatic Health Complaints; PHC, Psychosomatic Health Complaints; NDVI, Normalized Difference Vegetation Index; IQR, Interquartile Range; FAS, Family Affluence Scale. Source: Own elaboration.

Univariable models assessing the association between greenness and PHC (outcome variable) showed that, in boys, a one-IQR increase in greenness levels was associated with lower odds of presenting at least two psychosomatic health complaints more than once a week (OR 0.78, 95% CI: 0.63–0.98). After adjusting for age group, urbanization, and SES, the association was confirmed (OR 0.72, 95% CI: 0.53–0.98). The same association, although not statistically significant, was found in girls (OR 0.92, 95% CI 0.63–1.35) (Figure 1 and Supplementary material 1).

Considering PSYHC and SOMHC as separate outcomes, the results of the univariable, multilevel logistic regression models show that a one-IQR increase in greenness level was significantly associated with lower odds of presenting psychological health complaints in boys (OR 0.76, 95% CI: 0.60–0.95). After adjusting for age group, urbanization, and SES, the opposite association was confirmed among boys (OR 0.67, 95% CI: 0.50–0.92), while for girls, the association was not statistically significant (OR 0.91, 95% CI: 0.66–1.26). Somatic health complaints did not appear to be associated with the levels of greenness in the areas surrounding the schools (Figure 1 and Supplementary materials 2, 3).

## 4. Discussion

The present study aimed to assess whether the environment, specifically green spaces, could be associated with wellbeing in a sample of 11- and 13-year-old students living in Piedmont, a northwestern region of Italy. The study hypothesis was that higher exposure to greenness could be associated with improved wellbeing, measured as reported psychosomatic, psychological, and somatic health complaints. Our findings partially confirm our hypothesis: a higher exposure to greenness was associated with a lower occurrence of psychosomatic and psychological health complaints in boys but not with SOMHC. Previous studies on this topic have shown encouraging results (46): Ward et al. showed that higher green space exposure was related to greater emotional wellbeing among adolescents (47), and Feng et al. found that greenness, measured as the percentage of land use within each statistical area of residence covered by green space, was associated with parent-reported levels of mental wellbeing in 12- to 13-year-old students (48). Moreover, Wang et al. demonstrated that greenness, measured as the NDVI, was associated with a decreased risk of serious psychological distress in teenagers when considering the 350 m radius (49).

The association between exposure to greenness and lower psychosomatic and psychological health complaints might be linked to: (1) Higher chances of being engaged in outdoor physical activities among adolescents living closer to green areas. Physical activity has been shown to increase psychological wellbeing in adulthood as well as in children and adolescents (50). The relationship between physical activity and greenness has also been assessed by studies that demonstrate how the urban environment could influence the level of physical activity of citizens (51), showing a positive effect of living in a neighborhood with a high number of parks (52). (2) Green spaces in residential areas could offer meeting places to develop and maintain social bonds within the neighborhood, thus increasing social capital, defined as “features of social organizations, such as networks, norms, and trust that facilitate action and cooperation for mutual benefit” (53). In this regard, a recent systematic review demonstrated a strong link between green spaces and prosocial behavior among children and adolescents (54). (3) More green spaces can also lead to better air quality, which can have positive effects on health (55). This hypothesis was confirmed by Outdin et al.: air pollution [in terms of nitric oxide (NO_2_)] was associated with an increased risk of antipsychotic or sedative compound prescriptions in children and adolescents (56), and Bakolis et al. showed that long-term exposure to air pollutants (NO_2_, NOx, and PM2.5) is associated with mental disorders and physical health complaints indicative of mental distress (57).

The present study found a significant association between greenness and psychological health complaints in boys but not in girls. For females, the same association did not appear significant. Previous studies on this topic have shown mixed results (58, 59). Specifically, studies have noted that women frequent public parks less than men, which could further exemplify the differences between men and women in the association between greenness and psychological health complaints (59).

### 4.1. Strengths and limitations

The main strength of the study is the standardized, international protocol of the HBSC survey, which facilitates a high standard of data collection (32, 33). Another strength is the representativeness of the students of the Piedmont region. The novelty of the topic should also be mentioned, i.e., the association of wellbeing, measured as subjective somatic and psychological complaints, and greenness, measured using the NDVI, a validated instrument that “accurately reflects the amount of neighborhood greenness that can be observed directly by humans from the ground” (60). The measurement of greenness by the NDVI also reduces the potential bias of a self-reported perception of the amount of greenness by study participants, thus representing an objective measure of greenness.

The main limitation of the study is the self-reported nature of the survey. This could have led to the misinterpretation of some of the questions by the study participants, thus lowering the quality of the data. However, the HBSC survey is an international survey with a validated protocol and a high standard of data retrieval methods. Moreover, self-reported health complaints are reliable instruments for assessing the outcome of the present study (wellbeing) (4). Another limit is represented by NDVI calculated in a buffer radius of 1,500 m surrounding the schools attended by the students. This may reflect the presence of green spaces in the area surrounding the schools but not the neighborhood where the students reside. However, Italian secondary schools are usually widely and uniformly distributed within the whole territory and can therefore be assumed to be very close to their students' residences. Therefore, the NDVI of the 1,500-m radius could be considered a proxy of the greenness around the students' homes. This does not apply to Italian third-grade schools; thus, students aged 15 years (first year of third-grade school) were excluded from the overall sample.

### 4.2. Conclusions

In conclusion, the study points out that greenness could be associated with lower psychosomatic, especially psychological, health complaints in male adolescents. These results might be of interest to decision-makers with regard to promoting local policies aimed at building public parks and, more broadly, greener cities, through the implementation of policies that redesign the urban environment to increase green spaces, as suggested by the United Nations Sustainable Development Goals (target 11.7) (61). The results of the study also underline the need for interventions to detect and reduce the underlying factors that lead to gender differences in the relationship between greenness and the wellbeing of adolescents. Further studies are needed to confirm the findings of this paper.

## Data availability statement

The datasets presented in this article are not readily available because the data presented in this study are not publicly available according to the Italian HBSC data access policy. Requests to access the datasets should be directed to paola.dalmasso@unito.it.

## Ethics statement

The studies involving human participants were reviewed and approved by the Ethics Committee of the Italian National Institute of Health (Ref. PROT-PRE 876/17). Written informed consent to participate in this study was provided by the participants' legal guardian/next of kin.

## Author contributions

PL, PD, RB, and AB conceived of the presented project. VB, GSq, RB, PL, PD, and AB retrieved the data. GSc, GSq, VB, MB, PL, and PD analyzed the data. GSc, GSq, VB, MB, and AB wrote the paper. PL, PD, and RB critically revised the paper for important intellectual content. All authors contributed to the article and approved the submitted version.
