# Supplementary material for: The wellbeing of adolescents and the role of greenness: A cross-sectional study among Italian students
Source: Front Public Health. 2023 Jan 18;10:1050533. doi: 10.3389/fpubh.2022.1050533 (PMC9889974; doi:10.3389/fpubh.2022.1050533)
Supplement: Supplementary file 1 [file Table_1.pdf]

Supplementary Material

Supplementary Material 1. Psychosomatic perceived health complaints (at least two health complaints more than once a week). Univariable and multivariable multilevel regression models. Levels: schools / students.

**Males**									
		**Univariable**				**Multivariable**			
		**Odds Ratio**	**95% Confidence Interval**		**P value**	**Odds Ratio**	**95% Confidence Interval**		**P value**
NDVI 1500 (IQR)		**0.783**	**0.627**	**0.979**	**0.032**	**0.724**	**0.533**	**0.982**	**0.038**
Age category (13 years)		1.047	0.814	1.347	0.718	0.996	0.771	1.286	0.977
Urbanization	Low	ref				Ref			
	Medium	0.980	0.675	1.410	0.897	1.239	0.811	1.892	0.322
	High	0.834	0.538	1.292	0.418	1.258	0.711	2.227	0.430
FAS scale	Low	ref							
	Medium	0.790	0.574	1.087	0.148	0.824	0.598	1.136	0.238
	High	0.777	0.545	1.108	0.164	0.821	0.574	1.174	0.281
**Females**									
		**Univariable**				**Multivariable**			
		**Odds Ratio**	**95% Confidence Interval**		**P value**	**Odds Ratio**	**95% Confidence Interval**		**P value**
NDVI 1500 (IQR)		0.913	0.700	1.191	0.504	0.924	0.632	1.349	0.681
Age category (13 years)		**1.774**	**1.352**	**2.326**	**0.000**	**1.740**	**1.332**	**2.290**	**0.000**
Urbanization	Low	ref				Ref			
	Medium	0.986	0.640	1.518	0.950	1.080	0.641	1.821	0.771
	High	0.921	0.556	1.525	0.750	1.080	0.527	2.210	0.932
FAS scale	Low	Ref				ref			
	Medium	0.756	0.546	1.045	0.091	0.734	0.528	1.021	0.067
	High	0.901	0.620	1.311	0.587	0.874	0.596	1.281	0.489

Supplementary Material 2. Psychological perceived health complaints (at least two health complaints more than once a week). Univariable and multivariable multilevel regression models. Levels: schools / students.

**Males**										
		**Univariable**				**Multivariable**				
		**Odds Ratio**	**95% Confidence Interval**		**P value**	**Odds Ratio**	**95% Confidence Interval**		**P value**
NDVI 1500 (IQR)		**0.758**	**0.605**	**0.949**	**0.016**	**0.673**	**0.495**	**0.916**	**0.012**
Age category (13 years)		1.273	0.978	1.657	0.072	1.218	0.933	1.591	0.148
Urbanization	Low	Ref				ref			
	Medium	0.973	0.669	1.415	0.887	1.296	0.845	1.988	0.235
	High	0.869	0.556	1.360	0.540	1.429	0.803	2.542	0.225
FAS scale	Low	Ref				ref			
	Medium	0.846	0.607	1.178	0.323	0.901	0.645	1.259	0.540
	High	0.810	0.559	1.175	0.269	0.870	0.599	1.264	0.464
**Females**									
		**Univariable**				**Multivariable**			
		**Odds Ratio**	**95% Confidence Interval**		**P value**	**Odds Ratio**	**95% Confidence Interval**		**P value**
NDVI 1500 (IQR)		0.953	0.759	1.198	0.683	0.910	0.658	1.258	0.567
Age category (13 years)		**2.068**	**1.581**	**2.705**	**0.000**	**2.032**	**1.551**	**2.663**	**0.000**
Urbanization	Low	ref				ref			
	Medium	1.207	0.841	1.734	0.308	1.350	0.864	2.110	0.188
	High	1.061	0.691	1.630	0.786	1.264	0.682	2.343	0.457
FAS scale	Low	ref				Ref			
	Medium	0.890	0.650	1.218	0.467	0.864	0.626	1.191	0.372
	High	0.939	0.655	1.346	0.731	0.909	0.627	1.319	0.617

Supplementary Material 3. Somatic perceived health complaints (at least two health complaints more than once a week). Univariable and multivariable multilevel regression models. Levels: schools / students.

**Males**									
		**Univariable**				**Multivariable**			
		**Odds Ratio**	**95% Confidence Interval**		**P value**	**Odds Ratio**	**95% Confidence Interval**		**P value**
NDVI 1500 (IQR)		0.824	0.598	1.135	0.238	0.883	0.577	1.354	0.571
Age category (13 years)		0.847	0.578	1.242	0.396	0.804	0.546	1.186	0.272
Urbanization	Low	ref				ref			
	Medium	0.932	0.566	1.536	0.784	1.026	0.575	1.831	0.931
	High	0.674	0.357	1.271	0.223	0.806	0.359	1.810	0.602
FAS scale	Low	ref				ref			
	Medium	0.865	0.536	1.396	0.554	0.890	0.549	1.443	0.637
	High	0.943	0.557	1.594	0.825	0.976	0.573	1.662	0.929
**Females**									
		**Univariable**				**Multivariable**			
		**Odds Ratio**	**95% Confidence Interval**		**P value**	**Odds Ratio**	**95% Confidence Interval**		**P value**
NDVI 1500 (IQR)		1.065	0.832	1.362	0.616	1.05	0.749	1.468	0.782
Age category (13 years)		1.162	0.867	1.558	0.316	1.135	0.844	1.526	0.401
Urbanization	Low	ref				ref			
	Medium	0.921	0.624	1.358	0.677	0.880	0.558	1.389	0.584
	High	1.141	0.724	1.798	0.569	1.050	0.560	1.972	0.878
FAS scale	Low	ref				ref			
	Medium	0.836	0.586	1.193	0.324	0.820	0.573	1.172	0.277
	High	0.999	0.669	1.492	0.996	0.972	0.647	1.462	0.894
